# Benefits of Ginger and Its Constituent 6-Shogaol in Inhibiting Inflammatory Processes

**DOI:** 10.3390/ph14060571

**Published:** 2021-06-15

**Authors:** Iris Bischoff-Kont, Robert Fürst

**Affiliations:** 1 Institute of Pharmaceutical Biology, Goethe University Frankfurt, 60438 Frankfurt, Germany; i.bischoff@em.uni-frankfurt.de; 2 LOEWE Center for Translational Biodiversity Genomics (LOEWE-TBG), 60325 Frankfurt, Germany

**Keywords:** *Zingiber officinale* Roscoe, extract, 6-shogaol, inflammation

## Abstract

Ginger (*Zingiber officinale* Roscoe) is widely used as medicinal plant. According to the Committee on Herbal Medicinal Products (HMPC), dried powdered ginger rhizome can be applied for the prevention of nausea and vomiting in motion sickness (well-established use). Beyond this, a plethora of pre-clinical studies demonstrated anti-cancer, anti-oxidative, or anti-inflammatory actions. 6-Shogaol is formed from 6-gingerol by dehydration and represents one of the main bioactive principles in dried ginger rhizomes. 6-Shogaol is characterized by a Michael acceptor moiety being reactive with nucleophiles. This review intends to compile important findings on the actions of 6-shogaol as an anti-inflammatory compound: in vivo, 6-shogaol inhibited leukocyte infiltration into inflamed tissue accompanied with reduction of edema swelling. In vitro and in vivo, 6-shogaol reduced inflammatory mediator systems such as COX-2 or iNOS, affected NFκB and MAPK signaling, and increased levels of cytoprotective HO-1. Interestingly, certain in vitro studies provided deeper mechanistic insights demonstrating the involvement of PPAR-γ, JNK/Nrf2, p38/HO-1, and NFκB in the anti-inflammatory actions of the compound. Although these studies provide promising evidence that 6-shogaol can be classified as an anti-inflammatory substance, the exact mechanism of action remains to be elucidated. Moreover, conclusive clinical data for anti-inflammatory actions of 6-shogaol are largely lacking.

## 1. Introduction

Ginger (*Zingiber officinale* Roscoe) belongs to the Zingiberaceae family of flowering plants and is a very commonly used herbal spice due to its aromatic and pungent flavor. There are hints that the plant was medicinally used already about 5000 years ago, mainly in China and India [1]. The ginger rhizome has traditionally been applied against diseases such as cholera, cold, diarrhea, nausea and abdominal pain, lumbago, toothache, hemorrhage, hypertension, or the chronic inflammatory disease rheumatoid arthritis [1,2,3,4,5]. Moreover, in African folk medicine it has been used as carminative, diuretic, and antiemetic [6], and in Iranian traditional medicine it has additionally been used to treat ailments of the nervous system such as epilepsy, paralysis, or infarct [7].

The current, evidence-based therapeutic indication of the dried ginger rhizome (as a powdered substance)—attributed by the Committee on Herbal Medicinal Products (HMPC) of the European Medicines Agency (EMA) as “a well-established use”—is the “prevention of nausea and vomiting in motion sickness”. Moreover, it is used as “traditional herbal medicinal product for the treatment of mild, spasmodic gastrointestinal complaints including bloating and flatulence“ [5].

This review aims to elaborate and highlight anti-inflammatory actions of *Zingiber officinale* Roscoe and 6-shogaol, the main bioactive component in dried ginger rhizomes. In vitro and in vivo studies were comprehensibly outlined in order to provide the current state of affairs.

### Search Strategy

For the literature search, we used the databases PubMed, Google Scholar, and Web of Science. For searching relevant articles, we used the keywords “shogaol”, “*Zingiber officinale”,* “inflammation”, and “anti-inflammatory”. Furthermore, the reference list of all relevant publications was thoroughly searched for relevant articles within this topic. Studies performed with shogaol alone or comparative studies were considered as relevant. No publications were excluded due to publication date. We excluded studies when the applied concentrations or—for in vivo studies—the administered doses or application routes were not evident. We also excluded clinical studies without single or double blinding or suitable control groups. There were 30 relevant studies for shogaol, 25 for *Zingiber officinale,* and 8 that compared shogaol and gingerol.

## 2. Anti-Inflammatory Action of Ginger Extracts

The interest in studying pharmacological effects of ginger and its constituents on pathological conditions has grown within the last few decades. Recent studies suggest ginger-derived substances have effects not only on diabetes, asthma, metabolic syndrome and cancer [8,9,10,11], but also hold anti-inflammatory potential [4,12,13,14,15]. The first scientific reports on the anti-inflammatory actions of ginger go back to the 1980s.

In 1989, a study by Mascolo et al. [13] showed that the single oral administration of *Z. officinale* rhizome extract (extracted with 80% ethanol, unspecified drug extract ratio) at doses of 50 and 100 mg/kg reduced carrageenan-induced paw edema formation in rats by 22% and 38%, respectively. Of note, these results were comparable to the effects of acetylsalicylic acid [13]. More insight into the anti-inflammatory actions of ginger rhizomes was provided by Srivastava et al. [16,17]. Analyzing effects of aqueous extracts (extracted in organic solvents by thin-layer separation: n-hexane-ether-acetic acid [80:20:1, *v*/*v*] or chloroform-methanol-ethyl acetate [2:1:1, *v*/*v*] (drug extract ratio was not specified), among them extracts from ginger roots, they found that the ginger extract reduced levels of prostaglandins and thromboxane in isolated human platelets as well as their aggregation in vitro and inhibited the prostacyclin synthesis in rat aortae [16,17]. Moreover, Srivastava et al. [18] collected some hints that the ginger rhizome may be useful for the treatment of rheumatoid arthritis in humans: A case history of seven patients diagnosed with rheumatoid arthritis (males and females, aged 50–67 years) revealed that the consumption of 5 g of fresh or 0.5–1 g dried ginger over a period of three months resulted in pain relief, improved joint agility, and less stiffness [18]. Ahui et al. [12] employed an in vivo model of Th2-mediated airway inflammation in ovalbumin (OVA)-sensitized mice. The i.p. administration of 360 mg/kg of an aqueous ginger extract (extracted in water, 10% yield unspecified drug extract ratio) resulted in a reduced amount of eosinophils, neutrophils, and monocytes in the bronchoalveolar lavage fluid (BALF) and lung tissue [12].

Tumor progression is strongly associated with inflammatory processes. Cancer might arise from inflammation, and the tumor environment is surrounded by activated inflammatory cells [19]. A study by Habib et al. [14] demonstrates anti-inflammatory actions of ginger extract (ethanol extraction, unspecified drug extract ratio) in in vivo cancer models: immunohistochemistry staining of cervix cancer tissue sections of rats that were subjected to a choline-deficient diet revealed that the distribution of NFκB was much lower in ginger extract-treated (100 mg/kg; oral) animals compared to the control groups. Furthermore, immunohistostaining of NFκB revealed that the expression was significantly decreased in liver cancer-induced rats subjected to choline-deficient diet when a ginger extract was administered. In concert with this, the study showed that also the tumor necrosis factor (TNF) levels in breast cancer tissue and liver cancer cells of rats exposed to the diet were diminished by the ginger extract [14]. Moreover, a daily oral or intraperitoneal administration (50 and 500 mg/kg) of an aqueous *Z. officinale* extract (homogenized material in 0.9% NaCl extraction, unspecified drug extract ratio) over a period of 4 weeks in rats resulted in significantly reduced prostaglandin E_2_ (PGE_2_) serum levels after the application of 500 mg/kg of the extract (i.p. or orally) [20]. When administered orally, this effect was detectable already at 50 mg/kg. Oral application of a high dose of 500 mg/kg extract resulted in significantly attenuated levels of thromboxane B_2_ (TXB_2_) in the serum [20].

An aqueous ginger extract (extracted in water, liquid–material ratio 60:1) showed anti-inflammatory actions in vivo in a carrageenan-induced rat paw edema model [21]. The oral application of 25 up to 200 mg/kg significantly reduced the paw volume, and, therefore, increased edema inhibition in a dose-dependent manner. By analyzing important inflammatory mediators in edema exudates, the study demonstrated that the treatment with the extract resulted in significantly reduced levels of the eicosanoid PGE_2_ of inflammatory cytokines such as TNF, interleukin (IL)-6, IL-1β, and interferon (IFN) γ, or of the chemokine monocyte chemoattractant protein 1 (MCP-1), macrophage inflammatory protein (MIP), and CCL5/RANTES. Moreover, treatment with the extract reduced myeloperoxidase (MPO) activity in paw edema tissue samples and NO levels in paw edema exudates, while the total antioxidant capacity was significantly increased. Remarkably, this study used indomethacin (10 mg/kg) as control, and the highest dose of the extract exhibited similar effects to this widely used nonsteroidal anti-inflammatory drug (NSAID) [21].

In a murine collagen type II-induced rheumatoid arthritis model, the oral administration of an aqueous extract (extracted with water, yield 21.2% from 200 g root material) of *Z. officinale* (100 and 200 mg/kg) resulted in significantly reduced serum levels of the inflammatory cytokines IL-4, IFN-γ, and IL-17 [22]. In addition, IL-17 protein was measured in tissues of murine paws and spleens and was found to be significantly lowered in spleen while IL-17 protein was completely reduced to basal levels in paw tissue. Matrix metallopeptidases (MMPs) are known to be induced within the course of rheumatoid arthritis. This study demonstrated that MMP-1, -3, and -13 mRNA expressions were significantly reduced by the extract in paw tissue. Furthermore, the authors observed that by hematoxylin-eosin and safranin-O staining, administration of the extract strongly alleviated cartilage destruction and inflammatory cell infiltration. The study also analyzed the effects of *Z. officinale* extract in vitro on human primary synovial fibroblasts and demonstrated a significant reduction of IFN-γ and IL-17 mRNA and protein after pro-inflammatory activation of these cells with IL-1β. Additionally, IL-1β-triggered MMP-1, -3, and -13 mRNA levels were significantly reduced upon extract treatment [22].

In a study by Fouda et al. [23], the intraperitoneal administration of 50, 100, and 200 mg/kg *Z. officinale* extract (extracted with 70% ethanol, 1 mg of lyophilized material equivalent to 16.7 mg of fresh rhizomes) in a collagen-induced rat arthritis model reduced joint temperature and paw thickness and prolonged the paw removal latency. Serum levels of the cytokines IL-1β, IL-2, IL-6, and TNF, and anti-type II collagen antibodies as indicators for inflammatory actions were reduced. Of note, the application of 200 mg/kg was even more effective than the anti-inflammatory control compound indomethacin (2 mg/kg) [23].

A recently published clinical study by Dall’Acqua et al. [24] focused on pharmacokinetic analyses and immunomodulatory effects of a combined lipophilic extract of *Z. officinale* (25 mg) combined with 5 mg *Echinacea angustifolia* extract orally administered in softgel caspules. This single-blinded study was designed as follows: 10 healthy volunteers (6 male, 4 female, 30–55 years old); impairment of liver function; allergies; and intake of medications were precluded. The 12 h fasted volunteers received two softgel capsules before blood samples were drawn in time intervals up to 5 h. A microarray of peripheral blood mononuclear cells (PBMCs) from healthy volunteers involved in the study was performed. The volunteers were picked according to plasma profiles showing high, medium, and low absorption. 293 transcripts were up-regulated and 249 were down-regulated. Validation of microarray data by qPCR confirmed the up-regulation of PPM1B and RORA and the down-regulation of ALDOA, DEFA1, DEFA3, PRDX2, PRDX3, and SDCBP mRNA, indicating a shift of leukocytes to an anti-inflammatory phenotype. The immunomodulatory and anti-inflammatory effects were comparable to the impact of hydrocortisone (250 mg) [24].

In addition, a double-blind randomized controlled clinical trial [25] investigated potential effects of ginger powder (1 g, daily) on knee osteoarthritis in 120 participants. The results of this study pointed towards anti-inflammatory actions of ginger as cytokine (TNF, IL-1β) concentrations significantly dropped in plasma samples of patients after 3 months of ingestion. This study only provided first hints for anti-inflammatory actions of ginger powder in knee osteoarthritis, and follow-up studies providing effects on further inflammatory biomarkers have to be implemented. Nevertheless, these initial clinical studies are promising starting points for the elucidation of ginger and its main bioactive constituents for the actions as inhibitors of inflammation.

These studies provide first evidence of the anti-inflammatory impact of extracts from *Z. officinale* Roscoe rhizome.

## 3. Ginger Constituents

### 3.1. Chemical Composition of Z. officinale Roscoe rhizome

**Figure 1:**
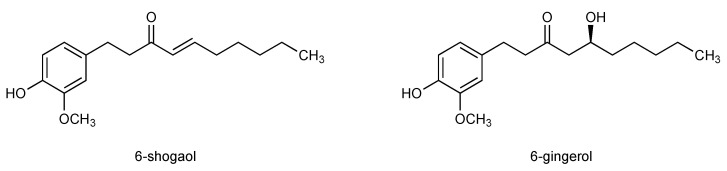
Chemical structures of 6-shogaol and 6-gingerol.

Already in the last decades of the 19th century, the ingredients of *Z. officinale* were being studied, and in the following century more than 400 ingredients of ginger were identified [15,26]. Among them, terpene compounds such as zingiberene, β-bisabolene, α-farnesene, β-sesquiphellandrene, and α-curcumene were found. Phenolic substances represent the pungent principles in ginger and make a great part of active compounds, among them gingerols (23–25%), paradols (5-deoxygingerols), and shogaols (18–25%) Gingerols and shogaols are the most prominent bioactive principles in ginger, among which are the main components 6-shogaol and 6-gingerol [15,27,28,29] (Figure 1).

### 3.2. 6-Shogaol, The Pungent Principle in Dried Ginger Rhizome

While 6-gingerol is the most bioactive compound in fresh ginger, 6-shogaol represents the main bioactive principle in dried ginger. The pungent compound 6-shogaol was identified and first described by Nomura in 1918. Here, shogaol was isolated by mild distillation of ginger rhizomes without contamination with gingerol [30]. About one decade later, Nomura and Tsurumi suggested the structural formula of “4-hydroxy-3-methoxyphenylethyl n-heptenyl ketone” for shogaol and synthesized the compound for the first time (Figure 1) [31]. Since then, a variety of methods to synthesize shogaols and active derivatives have been suggested [32,33,34]. Shogaols are vanilloids and contain Michael acceptor moieties; the electrophilic α,β-unsaturated carbonyl group reacts with nucleophilic substances (Michael donators). The presence of a Michael acceptor moiety is judged as an indicator for biological activity [35]. A kinetic stability study [36] using HPLC on 6-gingerol and 6-shogaol at temperatures ranging from 37 up to 100 °C at varying pH values (1, 4, 7) revealed that 6-gingerol underwent dehydration-hydration shifts with 6-shogaol. Gingerols are thermally instable and are easily transformed into shogaols. This study found that the degradation of 6-gingerol to 6-shogaol is strongly dependent on pH value. The greatest stability of gingerol was identified at pH 4 and a fast degradation was detected at pH 1 and a temperature of 100 °C. Under these conditions an equilibrium of 6-gingerol and 6-shogaol was adjusted after 2 h [37]. To gain deeper insight into the stability of 6-shogaol and 6-gingerol in the physiological situation, an in vitro study was performed mimicking gastric (pH 1) and intestinal (pH 7.4) conditions at 37 °C. Under gastric conditions, 6-gingerol and 6-shogaol implemented reversible dehydration and hydration to transform into one another, while this effect remained insignificant under intestinal conditions, which indicates relative stability of both compounds under physiological conditions at pH 7.4 [36]. 

## 4. Identification of Ginger Constituents Associated with Anti-Inflammatory Actions

Ginger has traditionally been used for the treatment of inflammation-associated diseases and a plethora of studies indicated its anti-inflammatory activity. Over the decades, the broad spectrum of ginger constituents and its main bioactive compounds have been identified. In addition, a number of studies analyzed the potential of those bioactive principles for anti-inflammatory actions. A review article by Kou et al. [38] outlined preclinical in vitro and in vivo studies that suggest 6-shogaol-derived actions against cancer, metabolic syndrome, pain, or nausea. Beyond the extensively investigated potential of 6-shogaol to inhibit cell growth in a large variety of cancer cell types, only a few studies investigated effects on the impairment of inflammation-related cell functions and signaling pathways in vitro and anti-inflammatory actions in vivo. 

In 1982, a cell-free in vitro study by Kiuchi et al. [39] identified constituents in ginger that inhibit prostaglandin synthesis by thin layer chromatography. A structure-affinity relationship study comparing the ginger constituents 8-paradol and 8-shogaol as well as the synthetic analogues 3-hydroxy-1-(4-hydroxy-3-methoxyphenyl)decane and 5-hydroxy-1-(4-hydroxy-3-methoxyphenyl)dodecane revealed inhibitory effects on cyclooxygenase 2 (COX-2) activity in the adenocarcinoma cell line A549 and determined an IC_50_ value of 2.1 µM for COX-2 inhibition by 6-shogaol [40]. Already in 1986, Flynn et al. found that pungent ginger constituents gingerdione and 6-shogaol are able to inhibit 5-lipoxygenase in human neutrophils indicated by inhibition of the formation of the eicosanoid 5-hydroxyeicosatetraenoic acid (HETE) and PGE_2_ [41]. 

A study by Wang et al. investigated the effect of orally administered ginger powder (200 mg/kg, suspended in 3 mL of an aqueous solution containing 1% carboxymethylcellulose), 6-gingerol (1 and 2 mg/kg), and 6-shogaol (0.5 and 1 mg/kg) on aspirin-induced gastric damage in rats [42]. While aspirin strongly induced the ulcers’ area, the additional administration of ginger powder completely blocked the formation of gastric mucosal lesions. Moreover, ginger powder abrogated the formation of ulcers with distorted gastric glands, damaged mucosal epithelium, and the formation of cell debris. Further analysis revealed that the administration of ginger powder resulted in the restoration of a normal physiological volume of gastric juice and acidity. Mucosal inducible nitric oxide synthase (iNOS) activity as well as TNF and IL-1β plasma levels were significantly reduced to control levels by the co-administrations of aspirin and ginger powder. The administration of the pure compounds 6-gingerol or 6-shogaol resulted in a strong and significant abrogation of aspirin-induced gastric ulcer formation. Similar to the effects of ginger powder, both compounds in combination with aspirin reduced iNOS activity and plasma levels of TNF and IL-1β [42]. These data suggest promising protective effects of the ginger powder and the pure compounds 6-shogaol and 6-gingerol against gastric ulcers.

By analyzing the effect of *Z. officinale* extract in the human keratinocyte cell line HaCaT, the study of Guahk et al. [43] revealed that the extract (0.1, 1 and 10 µg/mL) was able to significantly reduce UV-induced IL-1β, IL-6, IL-8, and TNF production. The treatment of HaCaT cells with the constituents 6-shogaol and 6-gingerol (0.1, 1 and 10 µM) showed similar results, whereas 6-shogaol exhibited stronger effects compared to 6-gingerol. Moreover, in a UVB-irradiated hyperplasia C57BL/6 mouse model, leukocyte infiltration as well as levels of IL-1β and IL-6 were strongly reduced upon *Z. officinale* extract treatment in a dose-dependent manner (1 or 2.5% extract, UVB treatment every alternate day, 14 days,) [43].

These insights into the actions of *Z. officinale* extract in vivo and in vitro demonstrate the benefits of ginger rhizome extract or powder on inhibition of inflammation-related processes. In particular, the in vivo studies employing important animal models highlight the potent inhibitory impact of ginger on the inflammatory phenotype on the one hand and pro-inflammatory mediators on the other hand (Table 1). The first insights that the bioactive principles of ginger, 6-shogaol, and 6-gingerol might be responsible for these actions are provided, while 6-shogaol seems to exhibit a greater anti-inflammatory impact.

Moreover, the aforementioned single-blinded clinical study with 10 volunteers by Dall’Acqua et al. [24] employing a combined lipophilic extract of *Z. officinale* combined with 5 mg *Echinacea angustifolia* found that 25 mg of the *Z. officinale* extract was comprised of 2.75 mg 6-gingerol and 0.75 mg 6-shogaol and 5 mg of *E. angustifolia* contained 0.5 mg dodeca-2*E*,4*E*,8*Z*,10*E*/*Z*-tetraenoic isobutylamide. Pharmacokinetic analysis revealed that 6-gingerol and 6-shogaol were found in plasma with a t_max_ of 58 and 95 min, respectively. C_max_ was determined for 6-gingerol with 5.66 ng/mL and for 6-shogaol with 9.25 ng/mL. These data indicate a rapid and high absorption of both *Z. officinale* ingredients after oral intake that might be responsible for anti-inflammatory actions [24].

## 5. Effects of 6-Shogaol on Inflammation-Related Processes

As comparative studies found higher impact of shogaols on a variety of pathological conditions, the interest in the anti-inflammatory actions of shogaols increased within the last decades. 

### 5.1. 6-Shogaol as Anti-Inflammatory Agent in Relevant In Vivo Models

In animal models, the treatment with ginger extracts or powder resulted in successful inhibition of inflammation-related symptoms and first insights about the phenomenologically actions were provided. Therefore, the interest in studying potential anti-inflammatory actions of the principle active constituent in dry *Z. officinale* rhizomes, 6-shogaol in vivo was brought into focus.

In 2006, an animal study by Levy et al. [44] demonstrated anti-inflammatory effects of 6-shogaol in a rat knee mono-arthritis model induced by complete Freud’s Adjuvant (CFA). A daily dose of 6.2 mg/kg 6-shogaol, 0.2 mL/day peanut oil (vehicle control) or indomethacin (2 mg/kg) as standard drug to demonstrate anti-inflammatory effects was administered orally via gavage one day before CFA injection over a period of 28 days. Edema swelling volume as well as number of total leukocytes, in particular lymphocytes and monocytes, were strongly and significantly lower than in control animals treated by peanut oil. These findings provide the first hints that 6-shogaol is, at least in part, responsible for the anti-inflammatory actions of ginger extract or powder. The vascular cell adhesion molecule 1 (VCAM-1) is one of the interaction partners of leukocytic integrins for diapedesis, and its soluble form represents a marker for inflammation. Analyzing blood samples of 6-shogaol-treated rats revealed a significantly lower concentration of soluble VCAM-1 compared to the peanut oil control [45]. As VCAM-1 is exclusively expressed in endothelial cells, we can conclude from this data that 6-shogaol might have a direct or indirect anti-inflammatory impact on the vascular endothelium.

In a rat passive cutaneous anaphylaxis (PCA) model, induced by the application of 2,4-dinitrophenyl hapten conjugated to human serum albumin (DNP-HAS), the oral application of 6-shogaol (1 and 5 mg/kg) resulted in a strong inhibition of PCA by 45 and 72%, respectively [46]. Further findings about the actions of 6-shogaol were provided using human mast cells that play a pivotal role in allergic inflammatory reactions and will be considered below. 

In a 12-*O*-tetradecanoylphorbol-13-acetate (TPA)-induced murine skin model, a single topical application of 1 and 2.5 µmol 6-shogaol reduced protein levels of iNOS and COX-2. Interestingly, they used 6-gingerol in the same study and the inhibitory effect on both proteins was much weaker compared to the treatment with 6-shogaol [47]. A couple of years later, the same group published a follow-up study showing similar results in a murine TPA-induced dorsal skin model [48]. Comparing the potential of 6-shogaol, 6-gingerol, and curcumin regarding their anti-inflammatory actions, they found 6-shogaol (1 and 2.5 µmol) to be the most potent compound in this setting. Moreover, dorsal skin samples were analyzed for IκBα degradation and phosphorylation as well as for nuclear NFκB subunits p50 and p65. 6-Shogaol demonstrated the strongest impact on reduction of IκBα degradation and phosphorylation as well as on attenuation of p50 and p65 protein levels in the nuclei over 6-gingerol and curcumin. Of note, only 6-shogaol blocked the TPA-induced ERK and Akt phosphorylation. Interestingly, the findings in this inflammation dorsal skin model demonstrate the impact of 6-shogaol on prominent inflammatory signaling cascades such as the NFκB, COX-2, and MAPK pathway in vivo [48]. As mentioned above, inhibiting effects on NFκB and reduction of PGE_2_ or TXB_2_ down-stream of COX-2 in vivo have been described for ginger extracts. This provides first evidence that 6-shogaol might be, at least in part, responsible for these actions. 

In a recently published study by Annamalai et al. [49], which employed a 7,12-dimethylbenz[a]anthracene (DMBA)-induced buccal pouch carcinogenesis hamster model, 6-shogaol reduced the inflammatory response. The animals either received 6-shogaol (20 mg/kg) alone or three times a week on alternate days to DMBA treatment over a period of 16 weeks. Western blot analysis of tissue samples revealed that 6-shogaol impaired the DMBA-induced activation of the NFκB signaling cascade, indicated by the down-regulation of IKK and p65 (cytosol and nucleus) protein levels. Moreover, DMBA-induced IκBα protein degradation was completely abrogated by the compound. Levels of NFκB subunit p65 mRNA were significantly reduced by 6-shogaol, assessed by agarose gel electrophoreses after PCR. Interestingly, using a model of cancer-related inflammation, 6-shogaol also shows inhibiting effects on the NFκB signaling cascade in vivo that correspond to the above mentioned findings of Levy et al. [45]. In the same study, the authors show the impairment of other inflammation-associated transcription factors down-stream of MAPK: The treatment with 6-shogaol resulted in a significant reduction of c-jun, c-fos protein levels and a significant down-regulation of AP-1 mRNA. In addition, protein levels of IL-6, IL-1, and TNF were almost blocked to control levels upon 6-shogaol treatment. Similar to the above reported findings of Pan et al. [47] 6-shogaol reduced the levels of COX-2 and iNOS in a DMBA-induced buccal pouch carcinogenesis hamster study [49]. 

In a recently published study employing an ischemic acute kidney injury (AKI) mouse model, 6-shogaol (20 mg/kg, i.p.) exhibited protective effects against renal ischemia-reperfusion injury by interfering with the NFκB and heme oxygenase 1 (HO-1) signaling pathways [50]. The main function of the HO-1 enzyme is to catalyze the oxidation reaction that degrades heme to ferrous iron. Protein levels of HO-1 were markedly increased in murine kidney tissue samples and the involvement of HO-1 in the actions of 6-shogaol was identified by the administration of the HO-1 inhibitor zinc protoporphyrin IX (ZnPP). While 6-shogaol was able to abrogate ischemia-reperfusion injury, the additional application of ZnPP repealed this protective effect. In addition, the inhibitory 6-shogaol-derived effect on neutrophil infiltration into renal tissue was abrogated by ZnPP. Moreover, 6-shogaol significantly reduced biomarkers of acute kidney injury: the levels of plasma creatinine, blood urea nitrogen, and mRNA of neutrophil gelatinase-associated lipocalin. Interestingly, ZnPP was again able to completely abolish these effects, indicating the involvement of HO-1 for the effects of 6-shogaol. The analysis of mRNA levels revealed that the inhibition of HO-1 suppressed the decreasing effect of 6-shogaol on IL-6, MCP-1, MIP-2, and keratinocyte chemoattractant (KC) levels upon ischemia-reperfusion injury. These interesting findings strengthen the hypothesis of the involvement of HO-1 and provide an important clue for the mechanism behind the anti-inflammatory and, moreover, protecting actions of 6-shogaol [50]. 

The neuroprotective effects of 6-shogaol were determined employing a murine model of middle cerebral artery occlusion (MCAO)-induced brain damage [51]. The daily oral administration of 6-shogaol (5 and 20 mg/kg) resulted in protection against transient focal cerebral ischemia, as indicated by a significant reduction of brain infarct volume and production of malondialdehyde (MDA) and of ROS after MCAO induction in murine brains. The analysis of IL-1β and TNF as well as COX-2 and iNOS revealed that their protein levels were significantly reduced upon 6-shogaol treatment. In addition, MCAO-induced ERK, JNK, and p38 activation was significantly reduced by both doses of 6-shogaol. Therefore, the authors of this study suggest a potential benefit of 6-shogaol for the prevention of stroke [51]. 

These findings in different in vivo models of inflammation demonstrate successful anti-inflammatory actions of 6-shogaol (Table 2), and most importantly, the benefit of this *Z. officinale* constituent over 6-gingerol, the bioactive principle in fresh ginger rhizomes. Important signaling pathways crucially involved in the process of inflammation such as NFκB, COX-2, or MAPK were successfully inhibited by 6-shogaol. Moreover, the engagement of the HO-1 for anti-inflammatory actions of the compound has been demonstrated.

### 5.2. 6-Shogaol Inhibits Inflammatory Processes In Vitro

In order to get an idea about the compound’s mode of action that stands behind the observed effects in the in vivo situation a plethora of approaches were performed in vitro (Table 3). In 2008 Pan et al. [47] demonstrated that 6-shogaol down-regulated the LPS-induced mRNA and protein levels and inhibited the activation of iNOS and COX-2 as indicated by reduced nitrite and PGE_2_ levels in RAW 264.7 cells after LPS induction. Most prominent effects were achieved at 10–20 µM without impairment of cell viability. Of note, the group also used 6-gingerol and found that 6-shogaol was a stronger inhibitor of COX-2 and iNOS than 6-gingerol. Focusing on the NFκB signaling cascade they found that 6-shogaol reduced both LPS-activated p50 and p65 translocation from the cytosol into the nucleus as well as NFκB activation in a reporter gene assay using RAW 264.7 cells. Further upstream, the degradation and phosphorylation of IκBα upon LPS stimulation was reduced by 6-shogaol. In concert with these findings, the group showed that the MAPK signaling was, at least in part, impaired by 6-shogaol. In particular, the activation of ERK was markedly reduced. Interestingly, the phosphorylation of p38 was not impaired by the compound. In addition, they found that the activation of the PI3K/Akt pathway in RAW 264.7 cells was inhibited by 6-shogaol. Of note, the effects of 6-shogaol on the NFκB, the MAPK, and PI3K/Akt cascade were detectable already at 6 and 10 µM [47]. These important findings correspond to the data obtained in in vivo experiments and provide even more information for the phenotypic actions of 6-shogaol. 

Similar results were obtained by Levy et al. The research group found that the inflammatory mediators TNF, IL-1β, and NO were significantly reduced in LPS-induced RAW 264.7 cells upon 6-shogaol (2, 10, and 20 µM) treatment [44]. A reporter gene assay showed that the activation of NFκB and COX-2 were significantly down-regulated by 6-shogaol (20, 30 µM) in LPS-induced RAW 264.7 cells, and the same impact was observed in HEK 293T cells when NFκB was activated via MyD88 or IKKβ [52]. In addition, in the murine hematopoietic cell line Ba/F3 and in HEK 293T cells the LPS-activated degradation of the kinase interleukin-1 receptor-associated kinase 1 (IRAK-1)—down-stream of the LPS receptor toll-like receptor 4 (TLR4)—was completely abrogated by the treatment with 20 and 30 µM 6-shogaol. For the induction of the NFκB signaling cascade the LPS-induced dimerization of TLR4 is inevitable. Immunoprecipitation experiments showed that LPS-induced TLR4 dimerization in Ba/F3 cells expressing TLR4-Flag (TLR4F), TLR4-GFP (TLR4G), MD2-Flag (MD2F), CD14, and NFκB luciferase was completely blocked when treated with 30 µM 6-shogaol [52]. Importantly, this study demonstrates the involvement of TLR4 for the actions of 6-shogaol in LPS-induced cells and the inhibition of the receptor dimerization might, at least in part, be responsible for the impact of the compound on the down-stream signaling. 

In a study employing human mast cells (HMC-1) [46], 6-shogaol strongly reduced the release of IL-6, IL-8, and TNF after activation of the cells by a combination of TPA and the divalent cation ionophore calcimycin (A23187) already at a concentration of 0.1 µM and almost blocked the release of these cytokines at 1 µM. Interestingly, nuclear NFκB levels and cytosolic IκBα phosphorylation were markedly reduced at 50 and 100 µM of 6-shogaol. Of note, in TPA- and A23187-activated HMC-1, 6-shogaol only showed significant inhibitory effects on JNK activation starting from 10 µM, while the level of ERK and p38 phosphorylation remained widely unimpaired. This observation is, at least in part, in contrast to findings of other studies that demonstrated a marked inhibition of ERK phosphorylation in RAW 264.7 cells or the inhibition of ERK, JNK, and p38 activation in the in vivo study by Na et al. [51] employing a MCAO-induced brain damage model. The varying effects on MAPK activity might depend on the cell type and model or the activation mode. Of note, 6-shogaol significantly affected the release of histamine from compound 48/80-activated rat peritoneal mast cells already at 0.1 µM [46].

A study using the TNF-activated human proximal tubular cell line (HK-2 cells) and LPS- or TNF-triggered isolated mouse proximal tubule cells revealed that increasing concentrations of 6-shogaol (50, 100, 150 µM) significantly reduced TNF, IL-6, IL-8, MCP-1, MIP-2, and ICAM-1 mRNA expression [50]. In HK-2 cells, TNF-induced nuclear translocation of the p65 subunit of NFκB as well as phosphorylation of IKK and IκBα was strongly reduced while IκBα degradation was prevented by 6-shogaol (150 µM). Importantly and similar to the above-mentioned findings in the AKI in vivo model, the protein levels of HO-1 were markedly increased by 6-shogaol. By using the p38 inhibitor SB203580, the study showed that the induction of the cytoprotective HO-1 in 6-shogaol-treated HK-2 cells was abrogated, and, therefore, mediated via p38 MAPK [50].

A study by Luettig et al. [53] demonstrated anti-inflammatory effects of 6-shogaol (100 µM) in the human intestinal cell line HT29/B6. The effect of 6-shogaol on cells that were exposed to TNF was analyzed by western blot, and results showed that the compound abrogated cytokine-induced phosphorylation of Akt, IκBα, and p65 subunit of NFκB. Of note, the analysis of the impact of 6-shogaol alone on the MAPKs p38 and ERK revealed that the compound, similar to the effects of TNF, induced the activation of both kinases, and 6-shogaol did not affect the TNF induced phosphorylation. Focusing on the TNF-induced barrier disruption of HT29/B6 cells, the study demonstrated that 6-shogaol exerts barrier protective effects: the compound increased transepithelial resistance (TER) values and prevented fluorescein (330 Dalton) permeability in a concentration-dependent manner (75, 100, and 125 µM). Immunofluorescence staining, qPCR measurement, and western blot analysis of the channel-forming tight junction protein claudin-2 revealed that 6-shogaol down-regulated the TNF-induced levels of claudin-2, while the levels of the sealing tight junction claudin-1 protein were significantly increased. These findings indicate a barrier-protective 6-shogaol-derived impact on the colon epithelial cell line. Importantly, an insight into the mode of action was provided by the application of the NFκB inhibitor BAY 11-7082, which resulted in a similar rise of TER values after a TNF-induced increase in barrier permeability. Interestingly, the PI3K inhibitor LY294002 did not mimic the effects of 6-shogaol in this experiment, which might imply that PI3K was not involved in the actions of 6-shogaol for barrier stabilization [53].

An in vitro study by Villalvilla et al. [54] employing human chondrocytes and the murine chondrogenic cell line ATDC5 demonstrated that the LPS- and IL-1β-triggered NO production was significantly decreased by 6-shogaol (5 µM). Interestingly, while the LPS-induced mRNA levels of MCP-1 and IL-6 were also reduced, treatment with 6-shogaol did not affect the IL-1β-activated gene expression levels of both cytokines. Interestingly, in this cell type, the LPS-activated phosphorylation of ERK was significantly down-regulated upon 6-shogaol treatment. In addition, to obtain an inactive compound with regard to anti-inflammatory actions, lacking the *α*,*β*-unsaturated ketone moiety, SSi6 was synthesized by the reaction of 6-shogaol with 2,4-dinitrophenylhydrazine, which forms a hydrazine with a carbonylic function of 6-shogaol. Indeed, SSi6-treatment of ATDC5 cells impaired neither the LPS- or IL-1β-induced NO production nor MCP-1 or IL-6 mRNA expression. The protein amount of adapter protein MyD88, which is widely used by TLRs to activate NFκB through IRAK, and iNOS were significantly reduced by 6-shogaol upon LPS-induced increase in protein levels, while the IL-1β-induced up-regulation of both proteins remained unimpaired by the compound. These findings are in concert with the results of the study by Ahn et al. [52] demonstrating that the dimerization of TLR4 and degradation of IRAK in LPS-induced Ba/F3 and HEK 293T cells was abrogated by 6-shogaol. 

The lysosomal cysteine protease cathepsin K is known to be involved in bone resorption and cartilage breakdown by degradation of elastin, collagen, and gelatin. Genetic deletion of cathepsin K has been shown to prevent inflammation and bone erosion in rheumatoid arthritis and periodontitis [55]. Indeed, Villalvilla et al. [54] demonstrated that the activity of cathepsin K was significantly reduced by 6-shogaol in human primary chondrocytes. Similar effects were observed when the modified synthetic derivative of 6-shogaol lacking anti-inflammatory properties, SSi6, was used, indicating that cathepsin-K inhibition is independent from an associated anti-inflammatory activity [54]. 

In addition, a study by Sang et al. [56] compared the effects of 6-gingerol and 6-shogaol on NO and arachidonic acid release in LPS-activated RAW 264.7 cells. The cells were exposed to radioactive arachidonic acid and an absorption of over 90% was assessed before the cells were treated with the compounds and LPS to induce release of radioactive arachidonic acid into cell culture media. The authors demonstrated that 6-shogaol (5 µM) significantly reduced the LPS-triggered exposition of arachidonic acid nearly to control levels, while up to 50 µM of 6-gingerol significantly but only slightly reduced the release. Comparing the compounds’ effects on NO synthesis in LPS/IFN-γ-triggered RAW 264.7 macrophages it was shown that the application of 5 µM 6-shogaol was superior in inhibition compared to 35 µM 6-gingerol [56].

These studies provide first insights into the anti-inflammatory actions of 6-shogaol and, moreover, demonstrate the superior potential of the compound in comparison to other ginger constituents. In addition, these investigations demonstrate the major impact of 6-shogaol on different stages of the NFκB pathway, independent of cell type or inflammatory stimulus. A number of studies used LPS for induction of an inflammatory cell response and it was demonstrated that already upstream of the NFκB signaling cascade the LPS receptor TLR4 was impaired by 6-shogaol. Therefore, the effects on the different stages of the down-stream pathway might be attributed to this effect.

### 5.3. Cell Protective Effects of 6-Shogaol against Inflammation-Related Oxidative Stress

Oxidative stress occurs due to an imbalance in antioxidant signaling pathways leading to the augmented formation of reactive oxygen species (ROS). The presents of ROS results in the onset of inflammatory signaling. The Nrf2/HO-1 signaling plays a pivotal role for the protection against oxidative stress. As mentioned above, in an in vivo AKI model and in vitro in HK-2 cells 6-shogaol increased the protein levels of HO-1 [50].

A study by Kim et al. [57] demonstrated that 6-shogaol was able to significantly reduce cellular oxidative stress in a concentration-dependent manner (1, 5, and 10 µM) in the H_2_O_2_-activated human hepatoma cell line HepG2. In concert with this, 6-shoagol treatment resulted in an increased amount of glutathione (GSH) in H_2_O_2_-induced HepG2 cells. The anti-oxidant response element (ARE) is present in promotor regions of gene that encode for detoxifying enzymes and cytoprotective proteins. In the situation of oxidative stress, the transcription factor nuclear factor erythroid 2-related factor 2 (Nrf2) dissociates from the Keap-1-Nrf2 protein-protein interaction, translocates into the nucleus, and binds to ARE. 6-shogaol (10 µM) increased the H_2_O_2_-triggered ARE activity and nuclear protein levels of Nrf2. Nrf2 plays a pivotal role in the event of oxidative stress within the inflammatory situation: Upon oxidative stress, it mediates a response that protects from cell damage and activates the transcription of HO-1 that exhibits a cell protective impact by removing ROS. Western blot analysis revealed that HO-1 protein levels were significantly increased by 6-shogaol in H_2_O_2_-activated HepG2 cells. As H_2_O_2_-triggered Nrf2 degradation was recovered by 6-shogaol, this study gives already a first clue about the intervention of 6-shogaol with the cellular inflammation-related oxidative stress response. Glutathione synthesis is mediated by the enzyme γ-glutamylcysteine synthetase (GCS). Western blot analysis showed that 6-shogaol was able to intensify the H_2_O_2_-induced upregulation of GCS protein levels in a concentration-dependent manner. In case of H_2_O_2_ activation, levels of phosphorylated JNK were significantly up-regulated in HepG2 cells upon 6-shogaol treatment. The additional application of the JNK inhibitor SP600125 abrogated the 6-shogaol-derived effects on the H_2_O_2_-induced up-regulation of JNK phosphorylation, nuclear Nrf2 levels, and of GCS and HO-1 protein. Moreover, the treatment of HepG2 cells with SP600125 attenuated the H_2_O_2_- and 6-shogaol-induced ARE luciferase activity and GSH levels. In addition, the anti-oxidative activity of 6-shogaol was reversed by the JNK inhibitor and the 6-shogaol-derived protein induction of HO-1, Nrf2, and GCS were abrogated by SP600125. These findings suggest the involvement of the JNK-Nrf2 signaling cascade for the actions of 6-shogaol [57].

6-Shogaol exhibits a stronger antioxidative activity than its homologues. Studies comparing bioactive ginger constituents demonstrated stronger antioxidant activity of 6-shogaol over 6-gingerol as demonstrated by Dugasani et al. [58]: 6-Shogaol induced the strongest effect on scavenging of 2,2-diphenyl-1-picrylhydrazyl (DPPH) in human polymorphonuclear neutrophils (PMNs) with an IC_50_ of 8 µM compared to the lower effectiveness of 6-gingerol (IC_50_: 26 µM). In addition, 6-shogaol was revealed to be the strongest scavenger of superoxide (IC_50_: 0.85 µM) and hydroxyl radicals (IC_50_: 0.72 µM) among the other analyzed ginger constituents. Moreover, *N*-formylmethionine-leucyl-phenylalanine (fMLP)-induced oxidative burst was reduced by 6-shogaol (6 µM) over a period of up to 10 min. In RAW 264.7 macrophages, 6-shogaol (1, 3, 6 µM) significantly reduced LPS-induced nitrite release in a concentration-dependent manner to a greater extent than gingerols. Similar effects were observed for the LPS-induced PGE_2_ release. Due to these findings and the identification of this strong variation of 6-shogaol-derived inflammatory and anti-oxidative effects compared to gingerols, the authors suggested the existence of an *α*,*β*-unsaturated ketone moiety in 6-shogaol for this prominent impact [58]. In addition, in the human keratinocyte cell line HaCaT, which was exposed to UVB light, 6-shogaol (20 µM) reduced COX-2 and iNOS protein levels as well as IL-6 and TNF release. In concert with the findings of Kim et al. [57] in HepG2 cells, 6-shogaol restored the UVB-induced down-regulation of the Nrf2 protein and mRNA in HaCaT cells. [59]. 

These findings shed light into the actions of 6-shogaol as anti-inflammatory compound by means of activating cell protective pathways via Nrf2/HO-1-JNK signaling.

### 5.4. The Impact of 6-Shogaol on the Inflammasome

The inflammasome represents a multi-protein complex of the innate immune system and its activation is associated with multiple diseases, such as type 2 diabetes, gout, or Alzheimer’s disease. The complex NLR family pyrin domain containing 3 (NLRP3) is one of the most relevant regulators in this process being controlled through its inhibitor Nrf2. Ho et al. [60] compared the bioactivity of 6-shogaol to gingerols and found that 6-shogaol exhibited a much stronger activity on canonical ATP- and LPS-triggered NLRP3 inflammasome activity in THP-1 macrophages. The study revealed that the application of 5 µM 6-shogaol reduced the IL-1β mRNA levels and protein secretion in THP-1-derived macrophages upon a combined treatment with LPS and ATP, while 20 µM 6-shogaol completely blocked the cytokine release. Similar results were obtained in experiments where TNF was used as pro-inflammatory activator. NLRP3 is predominantly expressed in macrophages and consists of the NLRP3 protein itself, the adapter protein adaptor apoptosis-associated speck-like protein containing a CARD (ASC), and caspase 1. The study demonstrated that 6-shogaol (20 µM) effectively inhibited total protein levels of NLRP3 and pro-IL-1β after a combined LPS and ATP-activated protein up-regulation. Of note, 6-shogaol reduced the increase of protein levels of LPS- and ATP-induced active caspase-1 [60].

This study provides interesting novel data of actions of 6-shogaol on the inflammasome of the innate immune system. As the mechanisms of the inflammasome are complex, further studies into the actions of 6-shogaol in this context should be acquired. Nevertheless, these data provide beneficial first insights that warrant for further research.

### 5.5. Inhibition of Neuroinflammatory Actions by 6-Shogaol

In 2012, Ha et al. [61] suggested anti-inflammatory actions of 6-shogaol in LPS-activated primary cortical neuron-glia cells from rats and the murine microglia cell line BV-2: 6-Shogaol (10 µM) was able to significantly reduce the levels of iNOS and NO as well as COX-2. In concert with this, the group also demonstrated a 6-shogaol-induced down-regulation of PGE_2_, IL-1β, and TNF. Moreover, 6-shogaol treatment of primary microglia cells resulted in a decrease of LPS-induced MAPK activity, as indicated by reduced levels of p38, JNK, and ERK phosphorylation. The NFκB activity was also reduced, since 6-shogaol reduced the NFκB DNA binding and abrogated the LPS-triggered IκBα phosphorylation and proteasomal degradation [61]. Similarly to Ha et al. [61] a study employing BV2 microglia cells [62] showed that 6-shogaol (5, 10 and 20 µM) significantly reduced the LPS-activated release of TNF, IL-1β, IL-6, and PGE_2_ in a concentration-dependent manner. In addition, the LPS-triggered NFκB phosphorylation and translocation into the nucleus was abrogated by 6-shogaol. In concert with this, IκBα phosphorylation was down-regulated, while its LPS-induced degradation was recovered by the compound. Interestingly, in this study they identified the involvement of peroxisome proliferator-activated receptor gamma (PPAR-γ) in the actions of 6-shogaol. This transcription factor regulates a plethora of genes, among them inflammatory cytokines such as IL-6 or IL-8, and shifts leukocytes to an anti-inflammatory phenotype [63]. Rising concentrations of the substance significantly increased the levels of the receptor, and the application of the PPAR-γ inhibitor GW9662 together with 6-shogaol and LPS reversed the inhibitory effect of 6-shogaol on TNF, IL-1β, PGE_2_, and IL-6 levels [62]. These data give rise to the hypothesis that the transcription factor PPAR- γ might be involved in the actions of 6-shogaol.

Another study focused on potential effects of 6-shogaol on the activation of histone deacetylases (HDACs) [64]. HDACs are suggested to exhibit anti-inflammatory actions by regulating the inflammatory gene expression [65]. An in vitro study by Shim et al. demonstrated potential anti-inflammatory actions of 6-shogaol (10 µM) in primary rat astrocytes, as the compound significantly reduced the LPS-triggered release of IL-1β and IL-6 release as well as the induction of iNOS and COX-2 protein. The LPS-caused induction of HDAC1 protein levels was reduced by 6-shogaol, and, in concert with this finding, the quantity of heat shock factor 1 and heat shock protein 70 (HSP70) were increased upon compound treatment. These effects were found to be similar to those evoked by the HDAC1 inhibitors MS-275 and trichostatin. An increase in HSP70, which is caused by the inhibition of HDAC, is accompanied by an increase in histone H3 acetylation. Indeed, 6-shogaol treatment resulted in a restoration of acetyl histone 3 protein after LPS degradation [64]. Due to these findings, it was suggested that 6-shogaol might play an important role for the inhibition of neuroinflammatory actions. These results are in line with the findings of animal studies by Na et al. [51] employing a MCAO-induced brain damage model demonstrating the inhibition of protein levels of iNOS, COX-2, and IL-1 β by 6-shogaol. In addition, the above-reported studies might give a hint about the involvement of HDAC1, PPAR-γ, as well as the NFκB signaling cascade for the 6-shogaol-derived inhibition of neuroinflammation. 

### 5.6. The Anti-Inflammatory Potential of 6-Shogaol Derivatives

As 6-shogaol appeared to be one of the most promising bioactive compounds in ginger, researchers made attempts to design and generate analogues. Gan et al. [66] synthesized 3-phenyl-3-shogaol and analyzed its impact on cancer cell invasion and inflammation in RAW 264.7 cells and in the invasive breast cancer cell line MDA-MB-231. Treatment of breast cancer cells with 3-phenyl-3-shogaol resulted in similar results as the application of 6-shogaol in RAW 264.7 macrophages. The application of 10 µM of the derivative suppressed different stages of the TPA-induced NFκB signaling cascade, as indicated by a significantly reduced promotor activity as well as IκBα degradation and phosphorylation. In addition, the TPA-triggered phosphorylation of IKK was strongly attenuated upon 3-phenyl-3-shogaol treatment. Moreover, the group showed that the derivative reduced p65 phosphorylation at Ser536, which is, at least in part, mandatory for the nuclear translocation of the transcription factor. In concert with this, 3-phenyl-3-shogaol reduced the NFκB promotor activity in LPS-induced RAW 264.7 macrophages. Moreover, secreted PGE_2_ was completely blocked by 10 µM 3-phenyl-3-shogaol treatment, and levels of COX-2, NO, and iNOS protein were significantly down-regulated [67]. The effects of 3-phenyl-3-shogaol in LPS-induced RAW 264.7 macrophages are comparable to the effects of 6-shogaol; the study by Pan et al. [47] demonstrated anti-inflammatory effects of 6-shogaol by the inhibition of NFκB subunit p65 phosphorylation, nuclear translocation, as well as reduced protein quantities of iNOS and COX-2 and attenuated levels of NO in LPS-activated RAW 264.7 cells.

A further approach focused on the development of an Nrf2 activator against oxidative stress. This attempt was inspired by 6-shogaol being known as beneficial inducer of Nrf2. An electrophilicity-based strategy led to the synthesis of (1*E*,4*E*)-1-(4-hydroxy-3-methoxyphenyl)-7-methylocta-1,4,6-trien-3-one (SA). This substance contains a conjugated unsaturated ketone chain. In vitro experiments using *tert*-butylhydroperoxide (*t*-BHP)-induced HepG2 cells demonstrated the beneficial upregulation of total and nuclear Nrf2 and induction of HO-1 protein. Moreover, the study showed that the 6-shogaol derivative SA (5, 10 µM) strongly inhibited Nrf2 ubiquitination and, therefore, led to the stabilization of the transcription factor. The ubiquitination of Nrf2 is strongly dependent on its up-stream regulator Kelch-like ECH-associated protein 1 (Keap 1). The authors of this study demonstrated a covalent modification of Keap 1 that finally leads to the inhibition of Nrf2 ubiquitination. Of note, the actions of SA were suggested to be carried out in a Michael acceptor-dependent fashion. The activation of ERK is inevitable for Nrf2 nuclear localization. Western blot analysis of the effect of SA on ERK phosphorylation revealed that the 6-shogaol derivative was able to strongly induce the activity of the MAPKs. Comparing the effects of 6-shogaol (10 µM) and SA (10 µM), the study demonstrated a superior impact of SA over 6-shogaol for Nrf2 activation, induction of ERK, and modification of Keap 1, which indicates a stronger cytoprotective impact. In addition, the application of SA resulted in higher levels of the detoxifying enzymes glutamate cysteine ligase catalytic subunit (GCLC) and glutamate cysteine ligase modulatory subunit (GCLM) as well as HO-1 [68].

## 6. Concluding Remarks

Since its discovery in the beginning of the last century, the actions of 6-shogaol, the major bioactive constituent in dried rhizomes of *Zingiber officinale* Roscoe, were extensively evaluated. Numerous in vitro and in vivo studies describe beneficial anti-cancer, anti-oxidant, and anti-inflammatory activities. In this review article, we mainly focused on the anti-inflammatory potential of 6-shogaol and presented a variety of studies that demonstrate the successful inhibition of inflammation. In vivo, 6-shogaol was able to significantly reduce hallmarks of inflammation, such as leukocyte infiltration or edema formation, and exhibited neuroprotective effects. Associated with anti-inflammatory actions in vitro, a study demonstrated 6-shogaol-derived barrier-protective effects in a colonic epithelial cell line. Interestingly, independent of the mode of inflammatory action, the application of 6-shogaol to different cell types or in in vivo models resulted in the successful inhibition of commonly known markers and signaling pathways of inflammation. 6-shogaol inhibited pro-inflammatory factors and mediators such as NFκB or COX-2, attenuated the levels of iNOS resulting in decreased levels of NO, and attenuated the release of pro-inflammatory cytokines such as interferon, TNF, interleukins, and chemokines. Moreover, 6-shogaol was able to promote protective effects by increasing the levels of Nrf2, which in turn resulted in an elevated quantity of HO-1 protein. This protective effect was most prominent when cells were exposed to cellular oxidative stress. Interestingly, dependent on the cell type or the mode of pro-inflammatory activation, contradictory results were obtained for the actions of 6-shogaol on the MAPKs p38, JNK, and ERK. Up to now, the reason for these varying results as well as the exact mode of action are unknown. A few studies suggest the involvement of PI3K/Akt, HDAC1, JNK-Nrf2 or PPARγ, and the inflammasome for the actions of 6-shogaol. These initial findings are still needed to be corroborated, and, thus, research in the elucidation of the mechanism of action of 6-shogaol is still ongoing. Although preclinical data regards the benefits of 6-shogaol as an anti-inflammatory compound, clinical studies within this context are largely missing up to now.

## Figures and Tables

**Table 1:** In vivo anti-inflammatory effects of *Zingiber officinale* Roscoe.

Organism/Model	Dose	Form	Administration Route	Effects
Rat/paw edema	50 and 100 mg/kg	extract	oral	Reduction of carrageenan-induced paw edema formation by 22 or 38%
Mouse/Th2-mediated airway inflammation in OVA-sensitized animals	360 mg/kg	extract	i.p.	Reduced amount of eosinophils, neutrophils, and monocytes in BALF and lung tissue
Rat/cervix cancer	100 mg/kg	extract	oral	Less NFκB distribution in tissue
Rat/liver cancer	100 mg/kg	extract	oral	Decreased NFκB expression
Rat/ breast cancer/liver cancer cells	100 mg/kg	extract	oral	Reduced TNF levels
Rat/saline administration	50 and 500 mg/kg	extract	oral or i.p.	Reduced PGE_2_ serum levels
Rat/saline administration	500 mg/kg	extract	oral	Reduced TXB_2_ serum levels
Rat/paw edema	25, 50, 100 and 200 mg/kg	extract	oral	Reduced carrageenan-induced paw volume, levels of PGE_2_, TNF, IL-6, IL-1β, IFNγ, MCP-1, MIP-2, RANTES, and MPO activity and NO levels
Rat/gastric ulcer	200 mg/kg	powder	oral	Block of aspirin-induced gastric mucosal lesion formation, abrogation of ulcer with distorted gastric glands, damaged mucosal epithelium, and the formation of cell debris Restoration of normal physiological volume of gastric juice and acidity; reduction of mucosal iNOS activity, TNF, and IL-1β plasma levels
Mouse/arthritis	100 and 200 mg/kg	extract	oral	Reduction of collagen II-induced IL-4, IFN-γ, and IL-17 protein and MMP1, 3 and 13 mRNA
Rat/arthritis	50, 100 and 200 mg/kg	extract	i.p.	Reduction of collagen II-induced joint temperature and paw thickness, serum levels of cytokines IL-1β, IL-2, IL-6, TNF, and anti-type II collagen antibodies, increased paw removal latency
Mouse/UVB-irradiated hyperplasia	1 and 2.5%	extract	oral	UVB-induced reduction of leukocyte infiltration, levels of IL-1β and IL-6

**Table 2:** In vivo anti-inflammatory effects of 6-shogaol.

Organism/Model	Dose	Administration Route	Effects
Rat/mono-arthritis model	6.2 mg/kg	oral	Reduction of edema swelling volume, lymphocyte and monocyte infiltration
Mouse/TPA-induced skin model	1 and 2.5 µmol	topical application	Reduction of iNOS and COX-2
Rat PCA model	1 and 5 mg/kg	oral	Reduction of DNP-HAS-induced PCA by 72% and 45%
Mouse/ischemic acute kidney injury model	20 mg/kg	i.p.	Involvement of NFκB and HO-1reduction of creatinine, blood urea nitrogen and mRNA of neutrophil gelatinase-associated lipocalin, neutrophil infiltration, andIL-6, MCP-1, MIP-2, and KC mRNA
Hamster, buccal pouch carcinogenesis model	20 mg/kg	oral	Reduction of DMBA-induced IKK, p65, COX, and iNOS levelsBlock of IκBα degradation and IL-6,IL-1 and TNFReduction of NFκB and AP-1 mRNA expression and c-jun, c-fos protein levels
Mouse, middle cerebral artery occlusion-induced brain damage model	5 and 20 mg/kg	oral	Reduction of brain infarct volume, MDA and ROS production, IL-1β, TNF, COX-2 and iNOS, ERK, JNK, and p38 activation

**Table 3:** In vitro anti-inflammatory effects of 6-shogaol.

Cell Type	Concentration	Effects
Human HaCaT cells	0.1, 1, and 10 µM	Reduced release of IL-1β, TNF, IL-6, IL-8
Human polymorphonuclear neutrophils	Increasing concentrations	DPPH scavenging: IC_50_: 8 µM
	6 µM	Reduction of fMLP-induced oxidative burst
Murine RAW 264.7 macrophages	1, 3, 6 µM	Reduction of LPS-induced nitrite and PGE_2_ release
Murine RAW 264.7 macrophages	5 µM	Reduction of LPS-triggered exposition of arachidonic acid and of LPS/IFN-γ-induced NO synthesis
Murine RAW 264.7 macrophages	2, 10, and 20 µM	Reduction of LPS-induced TNF, IL-1β, and NO
Murine RAW 264.7 macrophages	10–20 µM	Reduction of LPS-triggered mRNA, protein and activation of iNOS and COX-2; reduction of nitrite and PGE_2_
	6 and 10 µM	Reduction of NFκB nuclear translocation and IκBα degradation and phosphorylation; inhibition of ERK phosphorylation and PI3K/Akt activation
Human 293T cells	20 and 30 µM	Reduction of MyD88- and IKKβ-induced NFκB activity
Murine hematopoietic cell line Ba/F3	20 and 30 µM	Block of LPS-activated degradation of IRAK-1
	30 µM	Block of LPS-induced TLR4 dimerization
Primary rat cortical neuron-glia cells	10 µM	Reduction of LPS-induced NO, iNOS, COX-2 protein, PGE_2_, IL-1β, and TNF; inhibition of LPS-triggered p38, JNK and ERK phosphorylation and NFκB activity, IκBα phosphorylation and degradation
Murine microglia cell line BV-2	10 µM	Reduction of LPS-induced iNOS, COX-2
Human mast cells (HMC-1)	0.1 and 1 µM	Reduction of TPA/A23187-induced IL-6, IL-8, and TNF release
	50 and 100 µM	Reduction of nuclear NFκB and cytosolic IκBα phosphorylation
	10 µM	Inhibition of JNK activation
Rat peritoneal mast cells	0.1 µM	Reduction of compound 48/80-induced histamine release
Murine microglia cell line BV-2	5, 10, and 20 µM	Reduction of LPS-activated TNF, IL-1β, PGE_2,_ and IL-6 release, NFκB phosphorylation and translocation into the nucleus, IκBα degradation and phosphorylation; Increase of PPARγ
Human proximal tubular cell line HK-2	50, 100, and 150 µM	Reduction of TNF-induced TNF, IL-6, IL-8, MCP-1, MIP-2, and ICAM-1 mRNA; reduction of H_2_O_2_-induced IL-8, MIP-2, TNF, ICAM-1
	150 µM	Inhibition of TNF-activated nuclear NFκB, pIKK, pIκBα, and IκBα degradation
Primary mouse proximal tubule	50, 100, and 150 µM	Reduction of LPS/TNF-induced TNF, IL-6, IL-8, MCP-1, MIP-2, and ICAM-1
Human HepG2 cells	1, 5, and 10 µM	Reduction of H_2_O_2_-induced cellular oxidative stress; Increase of GSH, GCS, and ARE activity
	5 and 10 µM	Block of GST Upregulation of pJNK, Nrf2, and HO-1
Human THP-1 macrophages	5 and 20 µM	Reduction of LPS/ATP-triggered IL-1β and secretion and mRNA; inhibition of NLRP3 and active caspase-1
Human HT29/B6	100 µM	Reduction of TNF-induced Akt, IκBα and NFκB phosphorylation; induction of ERK and p38
	75, 100, and 125 µM	Increase of TER and prevention of fluorescein permeability and claudin 1, down-regulation of claudin 2
Murine chondrogenic cell line ATDC5	5 µM	Reduction of LPS/IL-1β-induced NO, LPS-induced MCP-1, IL-6, MyD88, ERK phosphorylation and iNOS
Human primary chondrocytes	5 µM	Reduction of cathepsin K activity
Primary rat astrocytes	10 µM	Reduction LPS-triggered IL-1β and IL-6 release, iNOS and COX-2 protein, LPS-induced HDAC1 protein and up-regulation of HSP70; restoration of acetyl histone 3 protein after LPS degradation

## Data Availability

Data is contained within the article.
